# Different Degree Centrality Changes in the Brain after Acupuncture on Contralateral or Ipsilateral Acupoint in Patients with Chronic Shoulder Pain: A Resting-State fMRI Study

**DOI:** 10.1155/2020/5701042

**Published:** 2020-04-25

**Authors:** Chao-Qun Yan, Jian-Wei Huo, Xu Wang, Ping Zhou, Ya-Nan Zhang, Jin-ling Li, Mirim Kim, Jia-Kai Shao, Shang-Qing Hu, Li-Qiong Wang, Cun-Zhi Liu

**Affiliations:** ^1^School of Acupuncture-Moxibustion and Tuina, Beijing University of Chinese Medicine, 11 Beisanhuan East Road, Chaoyang District, Beijing 100029, China; ^2^Department of Acupuncture and Moxibustion, Dongzhimen Hospital Beijing University of Chinese Medicine, Hai Yun Cang on the 5th Zip, Dongcheng District, Beijing 100700, China; ^3^Department of Radiology, Beijing Hospital of Traditional Chinese Medicine, Capital Medical University, 23 Meishuguanhou Street, Dongcheng District, Beijing 100010, China; ^4^School of Life Sciences, Beijing University of Chinese Medicine, 11 Beisanhuan East Road, Chaoyang District, Beijing 100029, China

## Abstract

Chronic shoulder pain (CSP) is the third most common musculoskeletal problem. For maximum treatment effectiveness, most acupuncturists usually choose acupoint in the nonpainful side, to alleviate pain or improve shoulder function. This method is named opposite needling, which means acupuncture points on the right side are selected for diseases on the left side and vice versa. However, the underlying neural mechanisms related to treatment are currently unclear. The purpose of this study was to determine whether different mechanisms were observed with contralateral and ipsilateral acupuncture at Tiaokou (ST 38) in patients with unilateral CSP. Twenty-four patients were randomized to the contralateral acupuncture group (contra-group) and the ipsilateral acupuncture group (ipsi-group). The patients received one acupuncture treatment session at ST 38 on the nonpainful or painful sides, respectively. Before and after acupuncture treatment, they underwent functional magnetic resonance scanning. The treatment-related changes in degree centrality (DC) maps were compared between the two groups. We found alleviated pain and improved shoulder function in both groups, but better shoulder functional improvement was observed in the contra-group. Increased DC in the anterior/paracingulate cortex and decreased DC in bilateral postcentral gyri were found in the contra-group, while decreased DC in the bilateral cerebellum and right thalamus was observed in the ipsi-group. Furthermore, the DC value in the bilateral anterior/paracingulate cortex was positively correlated with the treatment-related change in the Constant–Murley score. The current study reveals different changes of DC patterns after acupuncture at contralateral or ipsilateral ST 38 in patients with CSP. Our findings support the hypothesis of acupoint specificity and provide the evidence for acupuncturists to select acupoints for CSP.

## 1. Introduction

Shoulder pain is the third most common musculoskeletal problem after back and neck pain, with an annual prevalence of 20 to 50% [1, 2]. It is a major contributor to nontraumatic upper-limb pain. The disorder is typically characterized by the spontaneous onset of shoulder pain, shoulder stiffness, and a limited range of motion, which restrict activities of daily living [3, 4]. The clinical course of shoulder pain tends to be chronic and recurrence is common, with persisting symptoms for 6 to 12 months in 40 to 50% of patients [5]. Thus, chronic shoulder pain (CSP) imposes a considerable burden on the affected person and society.

Several conservative treatments have been advocated, including analgesics, nonsteroidal anti-inflammatory drugs, steroids, active physiotherapy, and health education [6]. Due to poor agreement on diagnostic criteria and the lack of specificity of clinical assessment that is considered a “gold standard”, no uniform, specific treatment protocol exists [7, 8]. Acupuncture, as a complementary alternative treatment, has been used in the treatment for CSP in China and has been also accepted for pain management in Western medicine [9]. The advantage of acupuncture for CSP is the lower incidence of adverse effects compared to pharmacy treatment [10]. Clinical randomized trials demonstrated that acupuncture has a short-term effect regarding pain and improving function for CSP [11–14].

According to the ancient and modern acupuncture books recorded, the Tiaokou (ST 38) was commonly used as a crucial distal acupoint to relieve the symptoms of shoulder pain. A high-quality trial with large samples, multicenter, and longer follow-up has observed that acupuncture in single acupoint ST 38 associated with physiotherapy was more effective in improving shoulder function and alleviating pain than physiotherapy as the sole treatment. The greater improvement also continued to increase during the 3 months after the treatment [15]. Base on the theory of Traditional Chinese Medicine, most acupuncturists usually choose contralateral acupoint for CSP treatment, which means acupuncture on the nonpainful side, to alleviate pain and to improve shoulder function of CSP. This method is named opposite needling. It refers to acupuncture points on the right side are selected for diseases on the left side and vice versa [16]. Many clinical trials have found that the therapeutic effect on shoulder pain by opposing needling at acupoint ST 38 is better than that by routine acupuncture treatment, such as in acupoints Jianzhen, Jianyu, and Jianliao [17, 18]. A systematic review suggests that acupuncture at contralateral ST 38 for shoulder pain achieved significant effects in improving the symptoms [16]. Unfortunately, its mechanisms of action are not well understood. In our previous study, by using the regional homogeneity (ReHo), we found the ReHo values in the anterior cingulate cortex (ACC) was found to be significantly more increased by treatment in the contra-group than that of ipsi-group. However, no correlation was found between ReHo in any brain region and clinical characteristics in each patient group by voxel-based correlation analyses [19]. Therefore, the underlying mechanisms with clinical evidence support are needed.

Over the past decade, resting-state functional magnetic resonance imaging (fMRI) has provided a valuable method to explore brain responses induced by acupuncture. Metrics that reflect regional spontaneous neuronal activity such as ReHo and the amplitude of low-frequency fluctuation (ALFF) have been developed. However, the brain is composed of dynamic and self-organized functional networks and hub regions that play pivotal roles in information flow in brain networks [20–22]. Unlike ALFF and ReHo techniques, voxel-wise degree centrality (DC) can quantify the importance of each node in the brain network and allows the mapping of functional integration in the brain at the voxel level [23]. The DC is an unbiased approach to detect changes in resting-state functional networks without selecting a particular region based on a priori hypothesis. Thus, the hub regions, which are involved in the mechanisms of contralateral acupuncture, can be revealed by using the DC method.

In the present study, we examined the difference of potential altered intrinsic functional hubs between acupuncture at contralateral acupoint ST 38 or ipsilateral acupoint ST 38 in unilateral CSP patients before and after a single treatment. The purpose of this study is to uncover the different neural mechanisms of contralateral and ipsilateral acupuncture at ST 38.

## 2. Materials and Methods

### 2.1. Participants

Patients with CSP were enrolled from December 2016 to July 2017 at the Beijing Hospital of Traditional Chinese Medicine Affiliated to Capital Medical University. The protocol was approved by the research ethical committee of the Beijing Hospital of Traditional Chinese Medicine affiliated to Capital Medical University (reference: 201315). Patients with CSP were recruited in outpatient clinics from the department of acupuncture. They were screened for enrollment by a face-to-face interview. All patients provided informed consent before participation.

Patients were considered eligible if they had the following criteria [19, 24, 25]: (i) left-sided shoulder pain for at least 6 weeks and no more than 1 year, (ii) age between 45 and 65 years, (iii) an average pain score of 50 mm or more on a 100 mm visual analogue scale (VAS) in the past week, (iv) the ability to communicate in Chinese, and (v) no history of shoulder surgery. Criteria for exclusion were as follows: (i) neurological disorders causing shoulder pain, (ii) referred pain from the cervical spine, (iii) osteoarthritis of the gleno-humeral joint or systemic bone and joint disorder (e.g., rheumatoid arthritis), (iv) receiving acupuncture or other current therapy involving analgesics in the preceding month, (v) overt psychiatric illness, (vi) drug or alcohol abuse, and (vii) structural MRI abnormalities in the brain and any contraindications for magnetic resonance imaging scans [26].

Twenty-four patients were enrolled after being screened for eligibility and were randomly allocated to the contralateral or ipsilateral acupoint groups. All patients underwent fMRI in baseline. Four patients in the ipsilateral group were excluded due to excessive head motion or not receiving the second fMRI scan.

### 2.2. Protocol

**Figure 1:**
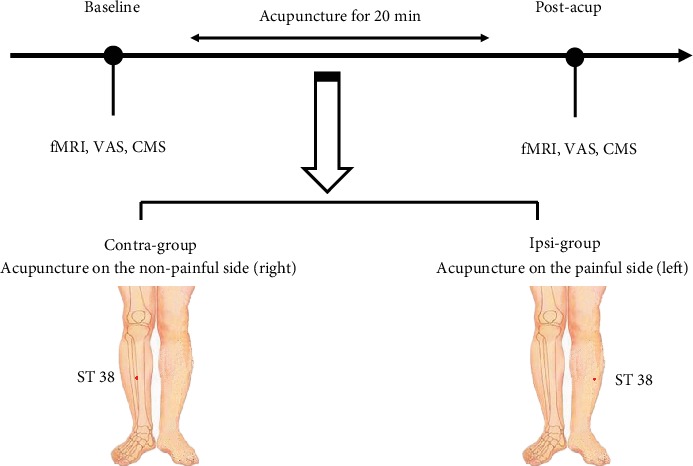
Study overview. Clinical assessments and fMRI were conducted at baseline and postacupuncture. Abbreviations: VAS: visual analogue scale; CMS: Constant–Murley score.

This study was a randomized, controlled trial by using fMRI scan to evaluate the effectiveness and mechanism of a single acupuncture session contralateral or ipsilateral to the CSP. The flow of the study included baseline fMRI scan, acupuncture treatment, and outcome fMRI scan (see Figure 1). Following baseline clinical and fMRI assessment, eligible patients were randomly assigned to one of two study arms (contralateral or ipsilateral acupuncture group). The acupuncture treatment lasted approximately 20 minutes. After a 5-minute interval, they underwent the 2^nd^ fMRI scan immediately. All procedures were completed in one day.

The randomization list was generated by use of the SPSS19.0 software and patients were randomly assigned to treatment groups in a 1 : 1 ratio. A researcher who has no other role in the study stored the randomization list and sight of the involved investigators. Randomized allocation of the next patient was concealed from the administrators and acupuncturist until the point of randomization. The patients, outcome assessors, and statisticians were blinded to treatment allocation. Patients were told that they will receive an effective intervention randomized after enrolment.

### 2.3. Acupuncture Treatment

Patients received a single acupuncture treatment between the two fMRI scans. For all patients, acupuncture needles were placed at contralateral or ipsilateral ST 38 based on their group assignment. Manual acupuncture was applied to achieve the Deqi sensation, which is a sensation of soreness, numbness, distention, or radiating that indicates effective needling. All acupuncture needles were single-use disposable needles, (Huatuo, Suzhou Medical Co. Ltd., Jiangsu, China). The patients were required to actively move their painful side shoulder with abduction, internal, and external rotation through the 20 minutes of acupuncture treatment.

### 2.4. Clinical Assessment

After the acupuncture treatment, all patients had a completed clinical assessment. The Constant–Murley score (CMS) is a 100-point scale that was used to evaluate shoulder function. Patients were asked to specify their pain level by indicating a position along a continuous line between 0 and 100 mm (0 mm indicated “no pain at all” and 100 mm indicated “worst pain imaginable”). Baseline characteristics, including sociodemographic characteristics, medication, and disease history, were also collected.

### 2.5. MRI Acquisition

At baseline and posttreatment, imaging data were acquired on a Siemens 3.0 Tesla scanner (Skyra, Siemens, Erlangen, Germany) with a standard headcoil in the Department of Radiology for the Beijing Hospital of Traditional Chinese Medicine Affiliated to Capital Medical University, China. The patients lay supine in the scanner with foam padding to minimize head movement and were instructed to close their eyes, keep awake, and move as little as possible. FMRI scans consisted of an echo planar imaging sequence using blood-oxygenation-level-dependent contrast: repetition time = 2000 ms, echo time = 30 ms, flip angle = 90 degrees, 33 slices, 3.5 mm slice thickness with a 0.6 mm gap, 220 × 220 mm in field of view, and 64 × 64 mm in plane resolution.

### 2.6. fMRI Data Preprocessing

The preprocessing of resting-state fMRI data was performed using SPM12 (Wellcome Department of Imaging Neuroscience, London, UK; http://www.fil.ion.ucl.ac.uk/spm) and DPARSF toolkit for MATLAB. The fMRI images were preprocessed in the following manner, including the removal of the first 10 images, time corrected, realigned, and head motion. Of note, three patients with more than 3 mm maximum displacement in *x*, *y*, or *z* and 3 degrees of angular motion were excluded in further analyses. The fMRI images were subsequently spatial normalized to the Montreal Neurological Institute (MNI) space. To further reduce the nuisance signals, Friston 24 head motion parameters, white matter, and cerebrospinal fluid signals were removed from the data via linear regression. Furthermore, a bandpass filter was performed within a frequency range of 0.01–0.08 Hz.

### 2.7. Voxel-Wise Degree Centrality Analysis

After the data preprocessing, the voxel-wise DC analysis was conducted using the REST software version 1.8 (http://www.restfmri.net). The rs-fMRI data analysis “REST-DC” toolkit (REST1.8; http://www.restfmri.net) was used to calculate binary DC measures according to the methods used in a previous study [26]. For each patient, whole-brain voxel-wise connectivity matrix was obtained by computing Pearson's correlation coefficient (*r*) between the time courses of one voxel with that of every other voxel within a predefined grey matter mask. This grey matter template has been released as a part of the tissue priors in SPM8 that included tissue with grey matter probabilities larger than 20%. In order to improve normality, each patient correlation matrices were transformed into a *z* − score matrix using Fisher's *r*-to-*z* transformation. We defined functional connectivity in the whole brain between a given voxel with every other voxel based on *r*_0_ = 0.25 correlation thresholds in binary version. In order to conform to the Gaussian distribution, the DC maps of each patient were then standardized by converting them to *z* − scores. The *z* − score transformation is achieved by subtracting the mean (DC of all voxels in brain mask) and dividing the standard deviation (DC of all voxels in brain mask). Subsequently, a smooth kernel of 6 mm full-width-at-half-maximum (FWHM) Gaussian kernel was applied to decrease spatial noise.

According to previous studies, five thresholds (*r*_0_ = 0.10, 0.15, 0.20, 0.25, 0.30, and 0.35) were used to compute DC in this study to avoid our primary results for the cortex to be dependent on the chosen threshold [26, 27]. Besides, the weighted version of DC was also computed.

### 2.8. Statistical Analysis

#### 2.8.1. Clinical Outcome Analysis

Data were reported as mean ± standard deviation (SD) for baseline and after treatment. Differences between patients' characteristics in the two groups before treatment were evaluated. Continuous variables of age, education, illness duration, VAS, and CMS with normal distribution were analyzed using independent *t*-test. The gender ratios were compared with chi-square (*χ*^2^) test. The VAS and CMS changes from baseline were assessed using paired *t*-tests. All statistical tests were performed in SPSS Statistics for Windows Version 22.0 (IBM Corporation, Armonk, NY, USA). Alpha will be set at level of 5%, and all statistical tests reported will be two-tailed.

#### 2.8.2. Degree Centrality and Brain-Behavior Correlation Analysis

The group difference, a comparison of the DC maps (post minus pretreatment), using an independent *t*-test (DPABI software package, http://www.rfmri.org) with whole brain mask was obtained. For each group, the DC maps were assessed using paired *t*-test. The significance level was set at a corrected *p* value < 0.05 with two-tailed. Multiple comparisons were corrected using the Gaussian random field (GRF) theory with DAPBI package.

Mean DC values of the obtained regions with significant group differences were extracted. Pearson's correlation analysis was performed to examine the association between the values of DC and the changes of VAS and CMS. All statistical analyses were performed using SPSS with a statistical significance level of *p* < 0.05.

## 3. Results

### 3.1. Demographics and Clinical Characteristics

Baseline and clinical outcome characteristics had previously been described in detail [19]. There was no significant differences in age, gender, and VAS and CMS scores between the contra-group and the ipsi-group at baseline (all *p* > 0.05).

Compared with pretreatment, pain and shoulder function significantly improved in both groups after treatment (all *p* < 0.05). A significant treatment difference was observed on CMS between the two groups [19]. The mean change from baseline in CMS after acupuncture treatment was 15.67 (SD 3.07) for the contra-group and 4.50 (SD 1.00) for the ipsi-group (*p* = 0.010, two-sample *t*-test). There was no significant difference in VAS between the contra-group (−20.42 ± 5.62) and the ipsi-group (−8.75 ± 2.27, *p* = 0.122, two-sample *t*-test).

### 3.2. Degree Centrality Difference between Patients in the Contra-Group and Ipsi-Group

**Figure 2:**
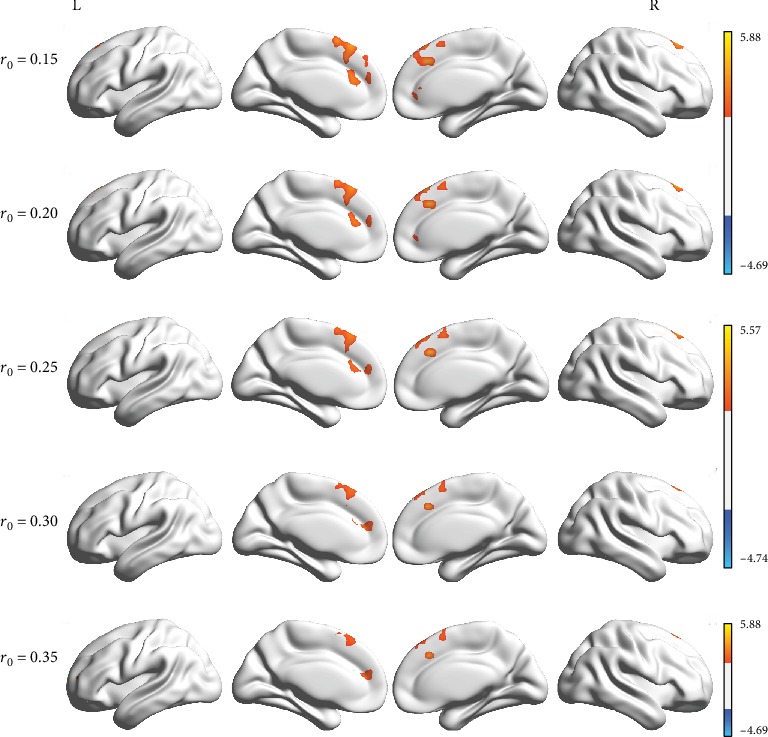
Group differences in the degree centrality (DC) between the contra-group and the ipsi-group before and after acupuncture treatment in binary version. Compared to the ipsi-group, the contra-group showed significantly similar increased DC according to different correlation thresholds (*r*_0_ = 0.15, 0.2, 0.25, 0.3, and 0.35). The effects are significant at a single voxel *p* < 0.05, GRF-corrected cluster level *p* < 0.05. The hot color indicates significantly increased DC brain area. Abbreviations: L: left; R: right.

**Figure 3:**
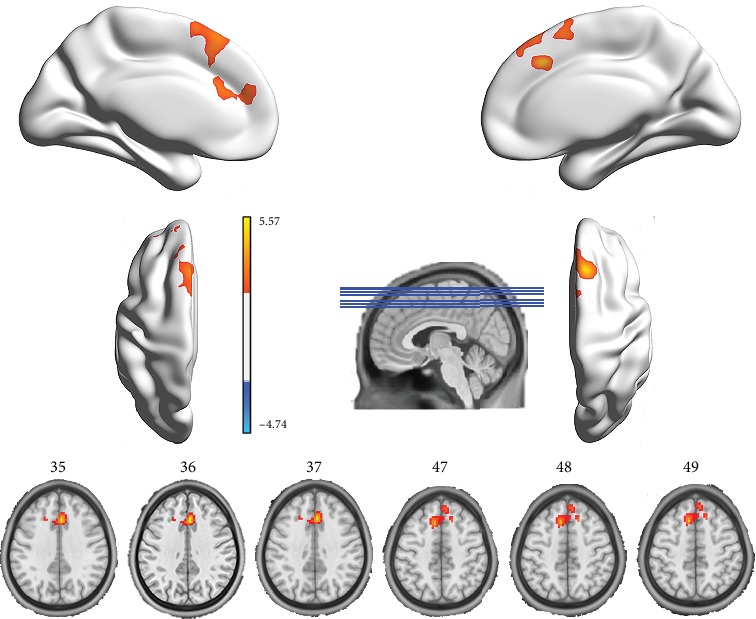
Group differences in the degree centrality (DC) between the contra-group and the ipsi-group before and after acupuncture treatment. Compared to the ipsi-group, the contra-group showed a significantly increased (marked in red) DC change in the anterior/paracingulate cortex. Abbreviations: L: left; R: right.

The DC maps changes pre- and posttreatment in the contra-group and ipsi-group were compared in several thresholds at *r*_0_ = 0.10, 0.15, 0.20, 0.25, 0.30, and 0.35. As shown in Figure 2, the functional hubs mainly localized in the anterior/paracingulate cortex and medial frontal gyrus (Supplementary Table 1). Besides, the weighted version of DC assuring the robustness of the findings with nearly identical results is shown in Supplementary figure 1. Therefore, the results of DC in correlation threshold at 0.25 in binary version were mainly reported in this study. Independent *t*-test results (CRF multiple comparisons corrected *p* < 0.05 and cluster size > 598 mm^3^) showed different DC in the bilateral anterior/paracingulate cortex (expand to superior frontal gyrus) between the two groups (Figure 3 and Table 1).

**Figure 4:**
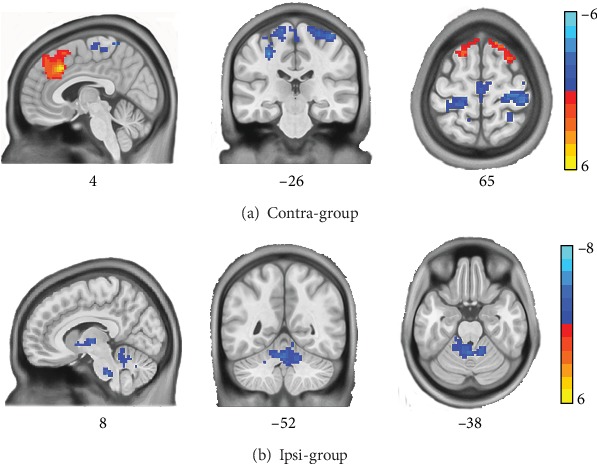
Significant changes in the degree centrality (DC) before and after acupuncture treatment in the contra-group and the ipsi-group. In the contra-group, significantly increased (marked in red) DC was found in the anterior/paracingulate cortex and decreased (marked in blue) DC in postcentral gyrus after acupuncture treatment (a). In the ipsi-group, significantly decreased (marked in blue) DC was found in the cerebellum (including the pons) and the thalamus after acupuncture treatment (b).

In the contra-group, paired *t*-test results (CRF multiple comparisons corrected *p* < 0.05 and cluster size > 411 mm^3^) showed that compared with pretreatment, acupuncture at contralateral ST 38 showed an increased DC in the bilateral anterior/paracingulate cortex (expand to superior frontal gyrus) and decreased DC in the bilateral postcentral gyri (Figure 4(a)). In the ipsi-group, compared with the pretreatment, acupuncture at ipsilateral ST 38 showed a decreased DC in the cerebellum bilaterally (including the pons) and the right thalamus with a paired *t*-test (CRF multiple comparisons corrected *p* < 0.05 and cluster size > 336 mm^3^, Figure 4(b)).

### 3.3. Correlation between DC and Clinical Variables

**Figure 5:**
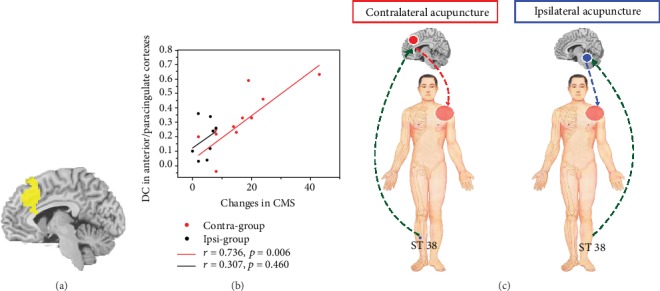
Correlation between the Constant–Murley scores (CMS) and degree centrality (DC) values in the anterior/paracingulate cortex and schematic summarizing CSP response to acupuncture treatment in contralateral or ipsilateral acupoint. (a) The anterior/paracingulate cortex mask with significant treatment-related DC changes in the contra-group. (b) Scatter plot between the treatment-related changes in CMS and DC in the contra-group and the ipsi-group. (c) Schematic of the different mechanisms of acupuncture at the contralateral and ipsilateral ST 38 for CSP. While acupuncture at the contralateral acupoint can improve shoulder function and alleviate pain via indirect regulated the anterior/paracingulate cortex, ipsilateral acupuncture can alleviate pain via the cerebellum. Red dots: contra-group; black dots: ipsi-group.

In the contra-group, Pearson's correlation analysis revealed that increased DC in the anterior/paracingulate cortex was positively correlated with the treatment-related CMS changes (*p* = 0.006, Figure 5). In the ipsi-group, the correlation analysis did not demonstrated a significant correlation between the changes from pretreatment in CMS/VAS and decreased DC in the bilateral cerebellum or right thalamus.

## 4. Discussion

We used resting-state fMRI to identify the spatial centrality distribution (hubs) and the connectivity density of the whole brain functional network in unilateral CSP patients and compared the DC maps between the patients who received acupuncture at the contralateral or at ipsilateral ST 38. The statistical analyses revealed that, compared to acupuncture at ipsilateral ST 38, patients in the contralateral group have great improvement in shoulder function and showed significantly increased DC in the anterior/paracingulate cortex. We also found that acupuncture at the contralateral ST 38 showed increased DC in both anterior/paracingulate cortexes and decreased DC in the postcentral gyri bilaterally. Acupuncture at ipsilateral ST 38 showed decreased DC bilaterally in the cerebellum and right thalamus. Furthermore, the DC value in the anterior/paracingulate cortex was positively correlated with the change from pretreatment in CMS in the contra-group rather than in the ipsi-group. These findings suggest different mechanisms for the effect of acupuncture at contralateral and ipsilateral ST 38. While acupuncture at the contralateral acupoint may improve shoulder function and alleviate pain via indirect effects on the anterior/paracingulate cortex, ipsilateral acupuncture may alleviate pain through the cerebellum.

The cingulate cortex belongs to the neural network underlying pain perception [28]. A meta-analysis demonstrated that regional gray matter decreases were consistently found in the paracingulate region bilaterally in chronic pain with fibromyalgia [29]. The structural changes might be a basis for impaired function. In chronic head pain patients, the paracingulate region was negatively connected to the dorsal insula [30]. Interestingly, a previous study showed that activation in the anterior cingulate cortex was evoked after acupuncture needle stimulation [31]. Here, we observed that acupuncture at the contralateral ST 38 acupoint showed an increased DC in anterior/paracingulate cortex, when compared with the ipsi-group. Our finding indicates that the anterior/paracingulate cortex might be an important hub affected by acupuncture stimulation at a contralateral acupoint. Additionally, the current study also demonstrated that increased DC in anterior/paracingulate cortex was positively correlated with the treatment-related CMS change in the contra-group. As a result, we suspect that acupuncture at the contralateral acupoint may improve shoulder function and alleviate pain via regulating the functional connectivity density between the anterior/paracingulate cortex and other brain regions. These observations are consistent with our previous study which found increased ReHo values in the anterior cingulate cortex in the contra-group compared with the ipsi-group [19]. However, the previous study should be regarded as exploratory in nature because no association of clinical features and the pattern of fMRI changes in the brain was observed. In the present study, we not only stressed the reliability of our previous theoretical basis but also provided a significant association with clinical features and provide further hints toward potential mechanisms of pain and function modulation by acupuncture stimulation at the contralateral acupoint.

We also observed that in the contra-group, decreased DC was found in the postcentral gyrus. It is the location of the primary somatosensory cortex (S1 cortex) and is part of the somatosensory system [32, 33]. The S1 cortex has been proposed to be involved in the localization and discrimination of pain, which directly receives painful sensation through the ascending nociceptive pathway, and abnormal functional and structural changes in the S1 cortex are also associated with chronic pain [34]. Animal and neuroimaging studies have consistently found enhanced brain activity and thicker grey matter volume in the S1 cortex during pain [34, 35], and those findings may support the possibility that the S1 cortex tend to be in an active state in response to pain. Acupuncture, as one of the oldest somato stimulus medical techniques for alleviating pain, may produce its healing efficacy by reversing this activation. In neuropathic pain rats, inflamed and amplified neural activities were observed in the S1 cortex after peripheral stimulation. Subsequently, the optical signals and region of activation in the S1 cortex were decreased substantially after electroacupuncture (EA) stimulation. The result implies that EA stimulation can have inhibitory effects on excitatory neuronal signaling in the S1 cortex, caused by noxious stimulation in neuropathic pain [36]. By using fMRI, Maeda et al. found that the brain response to verum EA produced deactivation in the ipsilateral S1 compared to sham EA in Carpal Tunnel Syndrome patients who reported reduced pain [37]. Our finding is partly consistent with previous studies and provides evidence that the S1 cortex may be involved in the underlying mechanism of acupuncture at contralateral acupoint for pain reduction.

In the ipsi-group, decreased DC was found in the cerebellum. The cerebellum is classically considered to be a brain region that participates in motor processing, but it has also been implicated in a number of integrative functions and neural processes beyond the motor domain [38]. Over the past decades, clinical, experimental, and neuroimaging studies, which investigated the role of the cerebellum in pain processing, have substantially increased. Animal experiments have shown that the cerebellum receives nociceptive inputs from cutaneous primary afferents [39] and nociceptive responses are affected by electrical or chemical stimulation of the cerebellum [40–42]. After cerebellar infarction, patients exhibited hyperalgesia when receiving heat and repeated mechanical stimuli, which suggests that the cerebellum plays an important role in pain perception and pain modulation in humans [43]. In fMRI studies, broad agreement has been reached about the contribution that cerebellar activation has seen in response to experimental nociceptive stimulation. A meta-analysis demonstrated that experimental and pathological pain both activate vermal lobule regions IV/V [44]. Interestingly, in the present study, the brain region, demonstrating a decreased DC after acupuncture at the ipsilateral acupoint, was in the cerebellum bilaterally overlapped with the vermal lobules IV/V. Similarly, Leung et al. demonstrated that changes in pain perception after acupuncture are associated with reduced functional activity in the cerebellum [45]. Our findings extended these observations obtained from measuring regional activity by calculating the full range of functional connectivity of the cerebellum to the whole brain, thereby illustrating a hub-like role of the cerebellum in pain relief using ipsilateral acupuncture for the first time. Furthermore, a change in DC in the pons also was observed in our study, and the cortical projections to the pons convey several different types of functional inputs that may pertain to pain [44]. Together, the current findings shed new light on the critical role of the hindbrain on the neural basis of acupuncture at ipsilateral acupoint for reliving pain.

Decreased DC in the right thalamus was found in the ipsi-group. The thalamus is a nuclear complex structure that serves as a relay of the cerebello-thalamo-cortical pathway, with nerve projection fibers connecting to cortical and subcortical brain regions [46]. Therefore, the thalamus has been viewed as a central relay station and integration center of the central nervous system. Recent work in nonhuman primates revealed the presence of cerebellar connections with the cerebral cortex via the thalamus [47]. The cerebellum is well positioned for modulating nociceptive processing, which has afferent and efferent connections with a wealth of cortical regions including some regions mediating endogenous pain modulation [48, 49]. The consistent changes of DC in the right thalamus and cerebellum in our study suggest that acupuncture at an ipsilateral acupoint may relieve pain through depressing the functional connectivity between the cerebellum and cortical regions via the thalamus. However, no DC change in cortical regions was observed in the ipsilateral group and future studies are needed to confirm it.

Acupuncture, as a stimulus of the surface of the body with inserting needles into the body, is effective in relieving pain. Many basic and clinical studies of acupuncture analgesia have suggested that acupoint-specific stimuli modulated the pain-related neuromatrix more effectively than placebo intervention [50]. This specificity suggests that acupuncture is more than a simple set of stimulus of the surface of the body but is focused on “specifically defined points” (i.e., acupoints), which are called acupoint specificity. According to the classical theory of Traditional Chinese Medicine, acupoint specificity is important and stimulation of different acupoints results in different effects [15, 51–53]. In our previous study, we found the presence of peripheral and central sensitisation at acupoints in participants with unilateral shoulder pain [54]. The recently developed technology, fMRI, has enabled a direct connection of the relationship between acupoint stimulation and neural activity in the human brain. Different brain responses were observed after acupuncture stimulation at contralateral or ipsilateral acupoint in the current study. DC changes in cortical regions were found in the contra-group, while in the ipsilateral group, DC changes in the cerebellum, pons, and thalamus were observed. Therefore, we suggest that the phenomenon of better shoulder functional improvement that was observed in the contra-group may be due to the effect of acupuncture at the contralateral ST 38 influencing the functional connectivity in hub cortical regions, while acupuncture at ipsilateral ST 38 regulates the cerebello-thalamo part of the cerebello-thalamo-cortical pathway. Our findings provide primary empirical evidence to confirm this acupoint specificity hypothesis.

The strengths of the present study include its control design and use of both clinical evaluation and fMRI as outcomes. Interestingly, the correlation between DC and clinical variables was found. Several limitations should be considered when interpreting the current findings. Firstly, because it was a pilot study with a sample size of only 20 patients and a type I error may have occurred. However, most of the clinical outcome measures observed showed significant and positive effects and statistical significance for the changes in the contra-group and ipsi-group, suggesting those brain changes were due to intervention effects rather than natural variability in the brain network. Besides, to reduce the false-positive outcomes, we performed multiple comparison correction required on the fMRI results. Future studies with larger sample sizes are needed to replicate the role of acupuncture in chronic shoulder pain. Secondly, the acupuncture treatment was performed and observed during a short period of time. Thus, it is unclear how long the brain changes related to acupuncture treatment at the contralateral or ipsilateral acupoint persist in chronic shoulder pain. However, our findings are consistent with a randomized controlled trial of subjects receiving contralateral acupuncture for 4 weeks, which demonstrated beneficial effects of contralateral acupuncture in the treatment of chronic shoulder pain, both in terms of pain and function [9]. Finally, although the purpose of this study is to uncover the different neural mechanisms of contralateral and ipsilateral acupuncture at ST 38, evaluating the mechanisms of bilateral acupuncture will help to understand the neural plasticity central function. Future studies with three groups are needed.

## 5. Conclusion

In this pilot study, we report different changes in the DC of brain after acupuncture at the contralateral or ipsilateral ST 38 acupoint in patients with chronic shoulder pain. Increased DC in anterior/paracingulate cortex and decreased DC in both postcentral gyri were found after acupuncture at the contralateral ST 38 acupoint. Decreased DC bilaterally in the cerebellum and right thalamus was observed after acupuncture at the ipsilateral ST 38 acupoint. Our findings support the hypothesis of acupoint specificity and provide evidence for acupuncturists to select acupoints for CSP.

## Figures and Tables

**Table 1:** Significant differences in degree centrality (*r*_0_ = 0.25) between the contra-group and the ipsi-group.

	Brain regions	Side	Condition	MNI coordinates			Cluster size	Peak *t* value
				*x*	*y*	*z*		
Differences between the two groups after treatment								
Contra-group minus ipsi-group^∗^	Anterior/paracingulate cortex	L R	Contra‐>ipsi‐group	3	24	39	900	5.57
Differences between before and after the treatment								
Contra-group	Anterior/paracingulate cortex	L R	Post > pre	3	24	39	1805	6.52
	Postcentral gyrus	L R	Post < pre	0	-45	69	710	-4.86
Ipsi-group	Cerebellum	L R	Post < pre	-12	-39	-30	416	-7.42
	Thalamus	R	Post < pre	24	-9	0	355	-9.56

Notes: ^∗^the result is achieved by comparing the means (post-DC values subtracted to the pre-DC values) of two groups. Abbreviations: L: left; R: right.

## Data Availability

The fMRI data used to support the findings of this study are restricted by the research ethical committee of the Beijing Hospital of Traditional Chinese Medicine affiliated to Capital Medical University, in order to protect patient privacy.

## Supplementary Materials

Supplementary Materials “Supplementary Figure 1: group differences in degree centrality (DC) between the contra-group and the ipsi-group before and after acupuncture treatment in weight version. The contra-group show remarkably similar altered DC brain areas (marked in red) according to different correlation thresholds (*r*_0_ = 0.15, 0.2, 0.25, 0.3, and 0.35) when compared to the ipsi-group. The effects are significant at a single voxel *p* < 0.05, GRF-corrected cluster level *p* < 0.05. Abbreviations: L: left; R: right.” “Supplementary Table 1: significant differences in degree centrality between two groups.”
