# Supplementary material for: Different Degree Centrality Changes in the Brain after Acupuncture on Contralateral or Ipsilateral Acupoint in Patients with Chronic Shoulder Pain: A Resting-State fMRI Study
Source: Neural Plast. 2020 Apr 25;2020:5701042. doi: 10.1155/2020/5701042 (PMC7197008; doi:10.1155/2020/5701042)
Supplement: Supplementary Materials — “Supplementary Figure 1: group differences in degree centrality (DC) between the contra-group and the ipsi-group before and after acupuncture treatment in weight version. The contra-group show remarkably similar altered DC brain areas (marked in red) according to different correlation thresholds (r0 = 0.15, 0.2, 0.25, 0.3, and 0.35) when compared to the ipsi-group. The effects are significant at a single voxel p < 0.05, GRF-corrected cluster level p < 0.05. Abbreviations: L: left; R: right.” “Supplementary Table 1: significant differences in degree centrality between two groups.” [file 5701042.f1.docx]

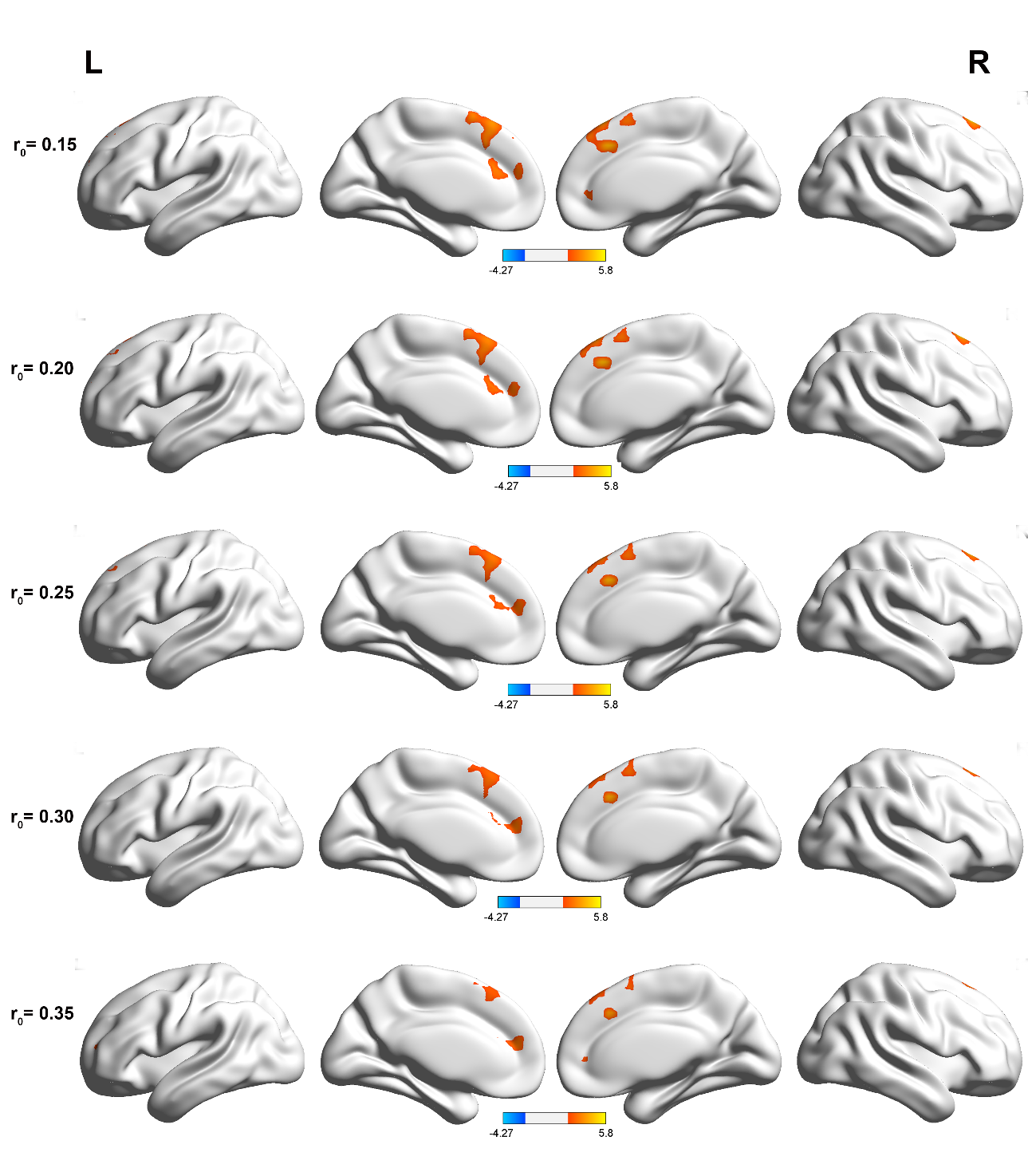


Supplementary Figure 1 Group differences in degree centrality (DC) between contra-group and ipsi-group before and after acupuncture treatment in weight version. The contra-group show remarkably similar altered DC brain areas (marked in red) according to different correlation thresholds (r_0_ = 0.15, 0.2, 0.25, 0.3 and 0.35) when compared to ipsi-group. The effects are significant at a single voxel p < 0.05, GRF corrected cluster level p < 0.05. Abbreviations: L: left; R: right.

Supplementary Table 1. Significant differences in degree centrality between two groups.

|  | **Brain regions** | **Side** | **Condition** | **MNI coordinates** | | | **Cluster size** | **Peak t value** |
| --- | --- | --- | --- | --- | --- | --- | --- | --- |
|  |  |  |  | **x** | **y** | **z** |  |  |
| **binary version** |  |  |  |  |  |  |  |  |
| r_0_ = 0.15 | Anterior/para cingulate cortex | L R | contra- > ipsi-group | 3 | 21 | 39 | 1078 | 5.88 |
| r_0_ = 0.20 | Anterior/para cingulate cortex | L R | contra- > ipsi-group | 3 | 24 | 39 | 988 | 5.93 |
| r_0_ = 0.25 | Anterior/para cingulate cortex | L R | contra- > ipsi-group | 3 | 24 | 39 | 900 | 5.57 |
| r_0_ = 0.30 | Anterior/para cingulate cortex | L R | contra- > ipsi-group | 3 | 24 | 39 | 891 | 4.44 |
| r_0_ = 0.35 | Medial Frontal Gyrus | L R | contra- > ipsi-group | -21 | -30 | 42 | 1458 | 4.22 |
| **weighted version** |  |  |  |  |  |  |  |  |
| r_0_ = 0.15 | Anterior/para cingulate cortex | L R | contra- > ipsi-group | 3 | 24 | 39 | 1029 | 5.80 |
| r_0_ = 0.20 | Anterior/para cingulate cortex | L R | contra- > ipsi-group | 3 | 24 | 39 | 947 | 5.71 |
| r_0_ = 0.25 | Anterior/para cingulate cortex | L R | contra- > ipsi-group | 3 | 24 | 39 | 895 | 5.29 |
| r_0_ = 0.30 | Anterior/para cingulate cortex | L R | contra- > ipsi-group | 3 | 24 | 39 | 829 | 4.61 |
| r_0_ = 0.35 | Thalamus | R | contra- > ipsi-group | 9 | -6 | 0 | 794 | 4.02 |
|  | Medial Frontal Gyrus | L R | contra- > ipsi-group | -21 | -30 | 42 | 1510 | 4.38 |

Abbreviation: MNI, Montreal Neurological Institute. Abbreviations: L: left; R: right.
